# *emm* type distribution pattern of group A streptococcus in north India: need for a new preventive approach

**Published:** 2010-12

**Authors:** V. Dhanda, R. Kumar, J.S. Thakur, A. Chakraborti

**Affiliations:** * Department of Experimental Medicine & Biotechnology; ** School of Public Health, Post Graduate Institute of Medical Education & Research (PGIMER), Chandigarh 160 012, India

Sir,

Group A streptococcus (GAS; *Streptococcus pyogenes*), pharyngitis in children assumes special significance due to the development of rheumatic fever (RF)/rheumatic heart disease (RHD). Streptococcal infections with an estimated death rate of over 500,000 individuals/year place GAS among major human pathogens, exceeded by HIV, *Mycobacterium tuberculosis, Plasmodium falciparum* and *S. pneumoniae*, and probably comparable to rotavirus, measles, *Haemophilus influenzae* type b, and hepatitis B^1^,^2^. Accurate characterization of GAS is required for better control of streptococcal diseases. GAS was classified on the basis of serotypic diversity of the M protein, a major virulence factor. The *emm* gene (encoding M protein) sequencing based on 5’ region^3^is becoming a universal method of choice. Though more than 180 *emm* sequence types and 800 *emm* subtypes have been described, new types/subtypes are still emerging from different regions^4^. GAS affects Indian population every year causing high cardiovascular mortality, morbidity leading to considerable socio-economic losses^5^.

Penicillin remains the only effective treatment against streptococcal diseases and no effective vaccine is available. Vaccine based on multiple amino terminal epitopes of GAS M protein that stimulates protective immunity against homologous strains^6^, do not provide broad coverage protection. Even the conserved regions of M type are being deciphered these days for the development of vaccines. Active epidemiological surveillance of GAS isolates in a north Indian region representing a particular ethnic population has been instituted to generate precise information on the transmission dynamics and *emm* type distribution for considering vaccine strategy in Indian scenario.

Carapetis *et al*^1^ estimated the occurrence of over 616 million incident cases of GAS pharyngitis worldwide per year. GAS pharyngitis and carriage in asymptomatic children varies in different countries. In India, prevalence of GAS pharyngitis and carriage ranges from 4.2 to 13.7 per cent and 1.3 to 20 per cent respectively^5^,^7^–^9^. GAS impetigo is quite common in south as compared to north India where pharyngitis is more common^9^. A few studies have reported the *emm* types from various geographical locations of India for a particular time span^7^,^9^–^12^. The *emm* types of these are heterogeneous in nature. Also the relationship of *emm* type with their isolation site needs to be discussed as has been done in the western world^13^. The types prevalent in India are different from those reported elsewhere^14^,^15^, suggesting that the same serotypes are present within a population, but vary between distinct geographical settings. In the absence of exact GAS type distribution data, true propensity of any preventive strategy against streptococcal infection remains incomplete. Moreover, for the eventual introduction and success of multivalent vaccines or vaccines based on conserved regions, better understanding of GAS infections and their molecular epidemiology in India becomes imperative.

We compiled the epidemiological surveillance data of GAS *emm* types carried out in some north Indian communities, *i.e*., Panchkula district of Haryana; Roopnagar of Punjab and Chandigarh during 1995 to 2007. The volunteers (school going children age 5-15 yr) from urban, rural and peri-urban slums were included in the study. A total of 17,071 samples were collected from north India during the study period. Throat swabs (n=14008) from peri-tonsillar and posterior wall of pharynx of pharyngitis patients or asymptomatic carriers, RF/RHD patients (n=11) and skin swabs (n=3063) from skin infected patients were taken. Betahaemolytic streptococcal colonies were isolated using standard protocol^16^, confirmed as Lancefield GAS by latex agglutination kit (Streptex, Murex Biotech Ltd, UK) and preserved in 20 per cent glycerol stocks in -70°C. Of these 17071 samples, 446 were confirmed to be GAS (276 from throat of pharyngitis /asymptomatic cases, 11 from RF/RHD and 159 from skin infected patients) with the prevalence rate of 2.6 per cent.

GAS isolates (240 from throat of pharyngitis/asymptomatic cases, 156 from skin infected patients and 11 from RF/RHD cases) were reconfirmed and *emm* typed by protocol as published earlier^7^; 373 isolates showed ≥95 per cent homology with the reference *emm* types, 16 isolates were identical to sequence types and 18 (4.4%) were M non typeable (MNT). The *emm* types were further designated as *emm* subtypes based on their variation from parent strain. A total of 70 types were obtained representing 53 known *emm* types with 33 subtypes, 12 sequence-types and five novel MNT strains (Fig.). *emm* 81 (15.7%) was the highly represented type common to both throat and skin infections. The seven most prevalent types- *emm* 81 (15.7%), 11 (7.9%), 112 (7.6%), 77 (7.4%), 44 (4.4%), 49 (3.7%) and 15 (3.4%) constituted 50 per cent of the isolates. Certain sequence types, like ST854, TP-c2135, AAB51153 and AAD 28609 recovered from Thailand and India, suggest a possible geographical bias in their isolation. MNT sequences - NS 292 and sp11014/VT-15, a rare type *emm* 1-2 and one new provisional sequence type (designated, StI129) were also identified. Most prevalent types found in GAS isolates from throat cultures were *emm* 81 (16.25%), 11 (10.8%), 77 (10%), 49 (4.6%), 75 (4.2%), 15 (3.3%), 71 (3.3%) and 68 (3.3%) as compared to *emm* types 81 (16%), 112 (15.4%), 44 (10.3%), 85 (5%), 15 and 1-2 (4.5%) from GAS skin cultures. These *emm* types constitute 56 per cent each of the throat and skin GAS cultures, respectively. Importantly, except for *emm* 81 and 15, other predominant types from throat infection were different from those in skin infections. Though several *emm* types were common to throat and skin sites, yet different types were seen in GAS cultures isolated from these sites, *e.g., emm* 1, 70, 80, 85, 104 and 5 sequence types were exclusively present in GAS skin cultures, *emm* 33 and 43 in GAS from RF/RHD patients and 33 different *emm* types were present only in throat GAS cultures. *emm* 1-2 was seen in both throat and skin GAS cultures. *emm* types 33, 43, 49, 74, 80, 93, 112 and NT were present in 11 RF/RHD cases. Hence, specific *emm* types can be associated with pharyngeal, skin and RF/RHD isolates.

**Fig. 1:**
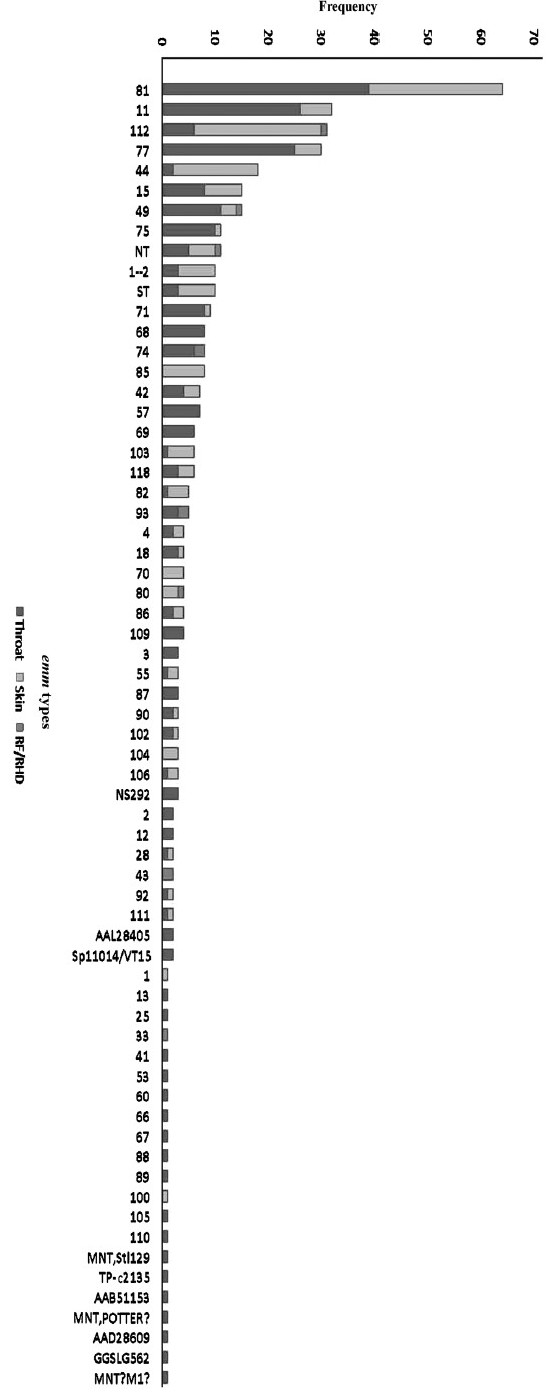
Distribution of *emm* types among north Indian group A streptococcus isolates.

On comparison with the epidemiological data from south, heterogeneity in GAS *emm* types was seen from both the regions. Of the 34 GAS isolates from south, 22 different types (64.7% diversity) were observed, the most common being *emm* 49; and out of 227 GAS isolates 59 *emm* types were seen with the most prevalent types being *emm* 112 (12.3%), 82, 11, 105, 108 and 110^11^,^12^,^17^. The first study on streptococcal pharyngitis patients from peri-urban slums near Chandigarh showed presence of *emm* types- 1-2, 11, 25, 49, 68, 69, 74, 75 and 90 with the most common being *emm* 49 and 75 (unpublished data). 59 GAS collected from pharyngitis patients visiting Chandigarh hospital and from villages of district Panchkula, Haryana during March 2000-2001 showed 33 different types with *emm* 74 to be most prevalent^7^. Later in 2000-2002, a total of 71 GAS cultures were collected from Raipur Rani, Panchkula, Haryana, that were deciphered into 17 different types, being *emm* 71 to be most prevalent followed by *emm* 81^11^. In year 2003, a total of 40 GAS when *emm* typed showed 23 different types with *emm* type 49 to be most prevalent in children from the slums, rural and urban areas of Chandigarh^10^. In 2002-2004, of the 139 GAS collected from rural community of Haryana, 27 different types were observed with *emm* 81 and 112 to be most common (unpublished data). Recent findings of GAS collected from rural areas of Punjab from 2002- 2007 depict *emm* 57 to be most prevalent (unpublished data). The pattern of *emm* type distribution varied from one region to another in north India with the passage of time. Earlier studies from north India showed high diversity of *emm* types encountered in a single year, but no specific *emm* type could be associated with the isolation site and those studies stressed upon the selection of *emm* types for vaccine development and/or need to tailor multivalent vaccine for regional use^7^,^10^. But the present observations indicate that the *emm* specific vaccine is not going to work in this region.

Surprisingly, only *emm* 11 was common amongst the six serotypes often seen in USA (*emm* 1, 3, 4, 11, 12, 28). We further looked for the types considered in the 26 valent multiple-epitope formulation vaccine and found though 50 per cent of those *emm* types (*emm* 1, 1-2, 2, 3, 11, 12, 13, 18, 28, 29, 33, 43, 75, 77, 89, 92) were seen in north Indian collection of *emm* types during the last 12 years; yet their frequency was quite less (around 15.4%) as compared to 80-85 per cent coverage of these types seen in western world^18^. The *emm* 81 is not a new *emm* type^19^ but predominated as an invasive strain in Sweden also^20^.

Reports examining the *emm* type diversity from developing nations support the presence of predominant types. In the Indian literature, only about 30 per cent of clinical GAS isolates were serologically M typed, therefore *emm* sequencing was initiated^21^. Our findings support the finding of heterogeneity among Indian strains. No marked preponderance of any single *emm* type was seen in South^9^,^11^,^12^,^17^. However, in north India substantial year-year, intra-site and inter-site variability in *emm* type distribution exists^7^,^9^–^11^, sometimes exceptionally striking, as compared to consistency found in other countries^18^. The individual GAS serotype enters and leaves a community quickly, however, *emm* 81 is seen prevalent throughout these years and also from both the north and south regions. Kaplan *et al*^22^ also reported displacement of M1 GAS with M6 in USA, further complicating the design of regional vaccines. The factors influencing the distribution of particular *emm* type are yet to be determined. Hence, it becomes important to monitor continuously the emerging *emm* types.

The strain variations have been noted within particular M types, and virulence has been linked with a particular isolate rather than being broadly related to a given serotype^23^. Hence *emm* types are not always adequate strain markers, because these can be shared by unrelated clonal types also. These observations would be of great significance and imply the efficacy of currently available vaccines may not be successful in India.

Pilot studies to check the protective efficacy of multivalent vaccine as well as considering a peptide sequence containing a conserved epitope from C-repeat region of M protein, were not encouraging^7^,^24^. The knowledge of circulating M types and molecular variants of other candidate antigens in the study population needs to be deciphered.

In conclusion, our findings like heterogeneous *emm* types encountered from north India, their association with isolation site and their divergence from western world are noteworthy. Regional and temporal variations in the prevalent *emm* types, emergence of new types and presence of M non-typeable strains in our country highlight better understanding of the present scenario and reveal an urgent need not only to reconsider the vaccine development but an alternative preventive approach to combat streptococcal diseases and their sequelae worldwide.
